# Development of a Recombinase Polymerase Amplification-Coupled CRISPR/Cas12a Platform for Rapid Detection of Antimicrobial-Resistant Genes in Carbapenem-Resistant *Enterobacterales*

**DOI:** 10.3390/bios14110536

**Published:** 2024-11-05

**Authors:** Ji Woo Yang, Heesu Kim, Lee-Sang Hyeon, Jung Sik Yoo, Sangrim Kang

**Affiliations:** 1 Division of Antimicrobial Resistance Research, National Institute of Health, Korea Disease Control and Prevention Agency, 187 Osongsaengmyeong2-ro, Osong-eup, Heungdeok-gu, Cheongju-si 28159, Republic of Korea; gosky0127@korea.kr (J.W.Y.); jungsiku@korea.kr (J.S.Y.); 2 DNA Analysis Division, National Forensic Service Busan Institute, 50 Geumo-ro, Mulgeum-eup, Yangsan-si 50612, Republic of Korea

**Keywords:** CRISPR/Cas12a, carbapenemase-producing *Enterobacterales*, recombinase polymerase amplification

## Abstract

The worldwide spread of carbapenemase-producing *Enterobacterales* (CPE) represents a significant threat owing to the high mortality and morbidity rates. Traditional diagnostic methods are often too slow and complex for rapid point-of-care testing. Therefore, we developed a recombinase polymerase amplification (RPA)-coupled CRISPR/Cas12a system (RCCS), a rapid, accurate, and simple diagnostic platform for detecting antimicrobial-resistant genes. The RCCS detected carbapenemase genes (*bla*_KPC_ and *bla*_NDM_) within 50 min, including 10 min for DNA extraction and 30–40 min for RCCS reaction (a 20 min RPA reaction with a 10–20-min CRISPR/Cas12a assay). Fluorescence signals obtained from the RCCS platform were visualized using lateral-flow test strips (LFSs) and real-time and endpoint fluorescence. The LFS clearly displayed test lines while detecting carbapenemase genes. Furthermore, the RCCS platform demonstrated high sensitivity by successfully detecting *bla*_KPC_ and *bla*_NDM_ at the attomolar and picomolar levels, respectively. The accuracy of the RCCS platform was validated with clinical isolates of *Klebsiella pneumoniae* and *Escherichia coli*; a 100% detection accuracy was achieved, which has not been reported when using conventional PCR. Overall, these findings indicate that the RCCS platform is a powerful tool for rapid and reliable detection of carbapenemase-encoding genes, with significant potential for implementation in point-of-care settings and resource-limited environments.

## 1. Introduction

Antimicrobials are essential tools for treating bacterial infections. However, antimicrobial resistance (AMR) has rapidly increased over the past few decades, and AMR infections may cause up to 10 million deaths by 2050 [1]. According to the previous study, AMR was associated with approximately 4.95 million deaths, with approximately 1.27 million deaths being directly attributed to bacterial AMR in 2019. This surpassed the mortality rates owing to other major infectious diseases, such as acquired immunodeficiency syndrome and malaria, which were estimated to cause approximately 860,000 and 640,000 deaths, respectively [2]. According to the World Health Organization (WHO), AMR is a crucial global issue, often referred to as a “silent pandemic”, and it is regarded as one of the top 10 health threats to humans [3].

Carbapenems are among the last-resort antibiotics to be used after clearly demonstrating the severity of resistant bacteria. In recent decades, the emergence and spread of carbapenemase-producing *Enterobacterales* (CPE) have become a significant public health concern because of their high transmission potential, which has led to endemic situations in several countries [4]. According to the Korea Disease Control and Prevention Agency (KDCA) Weekly Health and Disease Report, the incidence of carbapenem-resistant *Enterobacterales* (CRE) has been increasing annually. In 2022, CPE infections made up 71.0% of CRE infections, marking a 46.9% increase from 2021. CRE infections are increasing annually and becoming a significant social burden, thereby highlighting the need for epidemiological surveillance and continuous prevention and management efforts [5]. Carbapenemase production by CRE is the major mechanism underlying antimicrobial resistance. Carbapenemases are a type of β-lactamase that can hydrolyze carbapenem antibiotics. The primary carbapenemases found in *Enterobacterales* include *Klebsiella pneumoniae* carbapenemase (KPC), New Delhi metallo-β-lactamase (NDM), Verona integron-encoded metallo-β-lactamase (VIM), imipenemase (IMP), and oxacillinase-48 (OXA-48) [4,6]. Each of these enzymes possesses unique genetic and biochemical properties, which make the detection and management of CPE infections extremely complex [7]. Clinically, CPE infections are associated with high morbidity and mortality rates, contributing to a significant healthcare burden [8]. Treatment options are limited and often rely on old antibiotics with high toxicity profiles or new agents that may not be widely available or cost-effective [9]. Therefore, rapid and accurate detection of CPE is essential for effective infection control and patient management.

Rapid and accurate detection, which is the first and most crucial step to control AMR, typically relies on the identification of pathogen-specific markers such as nucleic acid sequences [10]. Molecular diagnostic methods for nucleic acid detection, including polymerase chain reaction (PCR), quantitative PCR (qPCR), and isothermal amplification, have become essential tools for identifying pathogenic genes [4]. PCR has been the only practical method for detecting trace amounts of infectious pathogens in biological samples. However, PCR assays require expensive equipment and skilled personnel to perform tests and interpret the results [11]. Unlike traditional PCR, isothermal amplification methods do not require costly thermal cycling equipment and can rapidly and efficiently amplify nucleic acids at a constant temperature. Several methods are used for isothermal nucleic acid amplification, including nucleic acid sequence-based amplification, signal-mediated amplification of RNA technology, loop-mediated isothermal amplification (LAMP), and recombinase polymerase amplification (RPA). Among these, LAMP and RPA are representative isothermal amplification methods, each with unique advantages and characteristics [12]. RPA operates at 37–42 °C and is an extremely sensitive and selective technology capable of amplifying even a minimal sample within 20 min [13]. Although nonspecific amplification may occur, the low reaction temperature, rapid amplification time, and simple operation without an initial denaturation step or the use of multiple primers make it ideal for simple and quick diagnostics [13,14,15].

The CRISPR system is a defense mechanism in bacteria and archaea against viruses and plasmid DNA. The system comprises CRISPR and its associated nuclease, Cas, and has been used for various purposes, including genome editing and pathogen detection [16]. Since the CRISPR–Cas system was first reported as a genome editing tool in 2012, it has been used for detecting nucleic acids in molecular diagnostics since 2016 because of its high sensitivity and low cost [11]. The recent advent of CRISPR technology has introduced a promising and innovative approach to rapid diagnosis [17]. The CRISPR/Cas system has been developed into various nucleic acid detection platforms, such as specific high-sensitivity enzymatic reporter unlocking (SHERLOCK), one-hour low-cost multipurpose highly efficient system (HOLMES), DNA endonuclease-targeted CRISPR trans reporter (DETECTR), and heating unextracted diagnostic samples to obliterate nucleases (HUDSON), in the diagnostic field [16,18,19,20]. These methods have been developed using various CRISPR/Cas effectors (Cas12a, 12b, 13a, and 13b) with *trans*-cleavage activity, i.e., collateral cleavage of untargeted sequences after the cleavage of the target (*cis*-cleavage). Cas12a recognizes the protospacer-adjacent motif (PAM) sequence (5′-TTTV-3′) within the target gene, binds to the target sequence via CRISPR RNA (crRNA), and subsequently cleaves it. Following the cleavage of the target gene, the untargeted *trans*-cleavage activity of Cas12a is initiated in the presence of a single-stranded DNA (ssDNA) probe that facilitates the generation of a detectable signal, such as fluorescence. Recently, to enhance the detection sensitivity before applying detection tools, nucleic acid preamplification has been commonly employed using methods such as isothermal amplification or PCR. This amplification procedure increases concentrations of the target gene, thereby improving detection sensitivity [21,22]. Therefore, the CRISPR–Cas12 system is frequently employed in conjunction with isothermal amplification techniques, such as RPA and LAMP, to enhance sensitivity. Notably, the RPA-integrated CRISPR/Cas12a system has been reported as a diagnostic tool for a range of pathogens, including severe acute respiratory syndrome coronavirus 2 (SARS-CoV-2), African swine fever virus, and methicillin-resistant *Staphylococcus aureus* [21,22,23,24].

Therefore, we aimed to develop a relatively rapid and accurate point-of-care detection platform for carbapenem-resistant genes prevalent in the Republic of Korea using RPA-coupled CRISPR/Cas12a systems.

## 2. Materials and Methods

### 2.1. Clinical Isolates and Confirmation of Carbapenemase Genes

In this study, the CPE strains were collected from general hospitals in the National Laboratory Surveillance System of the KDCA. A total of 24 CPE strains were used, including one non-CPE strain (*Escherichia coli* ATCC 25922), 13 strains of *K. pneumoniae*, and 11 strains of *E. coli*. Clinical isolates were identified using a MALDI Biotyper instrument (Bruker Daltonics, Bremen, Germany), and 16S rRNA sequencing was performed at a national reference laboratory. All strains were confirmed to have carbapenemase-encoding genes (*bla*_KPC_ and *bla*_NDM_), as previously reported [25].

### 2.2. Design of crRNAs and RPA Primers

The sequences of all subtypes of *bla*_KPC_ and *bla*_NDM_ were downloaded from the National Center for Biotechnology Information (NCBI) GenBank, and multiple sequence alignments were performed using ClustalW in BioEdit v.7.2. We selected the conserved regions of the sequence to align and design crRNA and RPA primers. First, the Cas12a PAM (5′-TTTV-3′) matching in conserved sequences were searched, and the corresponding two crRNA candidates were designed for optimal crRNA screening. The designed crRNA candidates were synthesized at GeneCker (Seoul, Republic of Korea). Based on the crRNA sequences, the corresponding RPA primers for amplifying each carbapenemase-encoding gene were designed using Primer3web. Specific parameters were set as follows: primer pair length between 28 and 35 bp, GC content between 30 and 70%, melting temperature between 30 and 70 °C, and amplicon length between 200 and 750 bp. The crRNAs and RPA primers are listed in Table 1 and Table 2.

### 2.3. Standard RPA Reaction

Using recombinant plasmids containing the target genes (*bla*_KPC_ and *bla*_NDM_) and genomic DNA extracted from clinical isolates as templates, a standard RPA reaction was performed using a TwistAmp Basic Kit (TwistDX, Maidenhead, UK), according to the manufacturer’s instructions. The reaction mixture contained 29.5 μL rehydration buffer, 2.4 μL each of 10 μM RPA primers, 1.2 μL DNA template, and 12.0 μL nuclease-free water. The components were thoroughly mixed and transferred to a reaction tube containing the lyophilized enzyme pellets. After the contents in the reaction tube were thoroughly mixed, 2.5 μL of 280 mM MgOAc was added and centrifuged. The RPA reaction was optimized using various conditions such as reaction time (10, 20, and 30 min) and temperature (37, 39, and 41 °C). The amplified RPA products were visualized using 2% agarose gel electrophoresis.

### 2.4. CRISPR/Cas12a Cleavage Assay and Detection of Fluorescence Signal 

A *cis*-cleavage assay mediated by LbCas12a (GeneCker, Seoul, Republic of Korea) was conducted using the RPA amplicons. The total volume of the CRISPR reaction mixture was 25 μL, consisting of 2.5 μL Rxn buffer (GeneCker) or 10× NEBuffer r2.1 (New England Biolabs, Ipswich, MA, USA), 2.5 μL RPA product, 175 nM LbCas12a, and 850 nM crRNA. We used 500 μM ssDNA probe (5′-FAM-TTATTATT-3′-BHQ) for fluorescence-based detection or FAM-Biotin ssDNA probe (5′-FAM-TTATTATT-3′-Biotin) (Integrated DNA Technologies, Inc, Hanam, Republic of Korea) for lateral flow-based detection.

The fluorescence signal was monitored using a real-time PCR instrument (Applied Biosystems, Foster City, CA, USA). The reaction proceeded at 37 °C for 30 min, and fluorescence intensity was recorded every 1 min. The endpoint fluorescence was visualized using a UV transilluminator (Bio-Rad Laboratories, Hercules, CA, USA).

Lateral flow test strip (LFS) detection was carried out using the HybriDetect Universal Lateral Flow Assay Kit (Milenia Biotech, Giessen, Germany). Briefly, Cas12a–crRNA (RnP) complex containing an FAM-Biotin ssDNA probe was mixed with RPA amplicons in a microcentrifuge tube, and the mixture was incubated at 37 °C for 20 min. Then, 75 μL HybriDetect Assay buffer was added, and a total of 100 μL mixture was incubated for 10 min at 37 °C. A HybriDetect strip was placed in the microcentrifuge tube, and the Control- or Test-line was visualized after 5 min. The relative intensity of the T-line was quantified using ImageJ and analyzed using GraphPad Prism v.8 (GraphPad Software, San Diego, CA, USA).

### 2.5. Optimization of the Trans-Cleavage Capability of CRISPR/Cas12a

To enhance the capability of *trans*-cleavage of the CRISPR/Cas12a system, we optimized the ssDNA probe concentration and ratio of crRNA to Cas12a. The ssDNA probe concentrations were set between 125 and 500 nM. The crRNA/Cas12a ratios were set; the ratios of 1:5, 1:4, 1:3, 1:2, 1:1, 2:1, 3:1, and 4:1, and the best combination was selected based on fluorescence intensity. The negative control was an RPA mixture without a DNA template, and fluorescence signals were recorded using a real-time PCR instrument. All experiments were repeated thrice.

### 2.6. Sensitivity and Specificity of the RPA-CRISPR/Cas12a System (RCCS)

To determine the limit of detection of the RCCS for *bla*_KPC_ and *bla*_NDM_, the DNA templates were serially diluted 10-fold (10 pg/µL to 10 fg/µL for KPC and 100 ng/µL to 100 pg/µL for NDM) using RNase-free water. RCCS-based reactions were performed using the diluted DNA templates.

The specificity of the RCCS was evaluated using genomic DNA samples of carbapenemase-producing *K. pneumoniae*, which included six types of carbapenemase-encoding genes (*bla*_KPC_, *bla*_NDM_, *bla*_OXA-48_, *bla*_IMP_, *bla*_GES_, and *bla*_VIM_), and a non-CPE strain without a carbapenemase gene as templates. The bacterial strains were cultivated overnight at 37 °C in TSB broth (BD Difco, NJ, USA), and their genomic DNA was extracted by boiling at 95 °C for 5 min using a PowerPrep^TM^ Quick DNA Extraction Kit (KOGENEBIOTECH, Seoul, Republic of Korea). RCCS-based reactions were conducted under the optimized reaction conditions. All experiments were repeated thrice.

### 2.7. Evaluation of the RCCS Platform for the Clinical Isolates

To confirm consistency, the RCCS platform was compared with conventional PCR using clinical isolates. The PCR mixture included 5 μL of 5× Q5 Reaction Buffer, 0.5 μL of 10 mM dNTP, 0.5 μM each of forward and reverse primers, 1 μL template, and 0.02 U/μL Q5 High-Fidelity DNA Polymerase, and the volume was made up to 25 μL using double-distilled water. The PCR cycling program consisted of initial denaturation at 98 °C for 30 s, followed by 35 cycles of denaturation at 98 °C for 10 s, annealing at 56 °C for 30 s, and extension at 72 °C for 20 s, and a final extension at 72 °C for 2 min. Subsequently, the amplification products were analyzed using 2% agarose gel electrophoresis.

### 2.8. Determination of the Target Genes in Urine Sample

To determine the *bla*_KPC_ and *bla*_NDM_ genes from the urine samples, the bacteria at different concentrations (10^8^–10^3^ CFU/mL) were inoculated into synthetic urine (TMALAB, Seoul, Republic of Korea). Synthetic urine without the addition of any bacteria was used as a negative control. Urine samples with added bacterial solution were boiled at 95 °C for 5 min, and the supernatant was used as a template. RPA amplification and CRISPR/Cas12a cleavage analysis were conducted following the RCCS workflow [26].

### 2.9. Statistical Analysis

All experiments were independently performed at least thrice. Band densities were quantified using ImageJ, and statistical analyses were performed using GraphPad Prism v.8. The results are expressed as mean ± standard deviation (SD), and differences were considered significant at *p* < 0.05.

## 3. Results

### 3.1. Workflow of RCCS-Mediated Detection of bla_KPC_ and bla_NDM_

**Figure 1:**
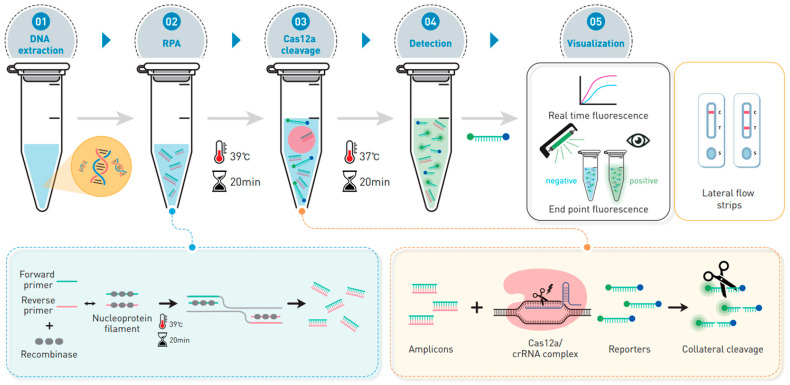
Schematic overview of RPA-CRISPR Cas12a/crRNA (RCCS) assay for rapid detection of carbapenemase genes. RPA was used for amplification of *bla*_KPC_ and *bla*_NDM_ genes. The RPA products were subsequently added to the CRISPR/Cas12a system that can specifically recognize *bla*_KPC_ and *bla*_NDM_ genes, respectively. Finally, the results can be interpreted using real-time PCR and UV trans-illuminator or LFS.

The principle of RCCS-mediated detection of *bla*_KPC_ and *bla*_NDM_ is shown in Figure 1. Initially, genomic DNA extracted from clinical isolates or plasmid DNA was amplified using the RPA reaction. Subsequently, the RPA products were introduced to the CRISPR/Cas12a detection system, which specifically recognizes and cleaves *bla*_KPC_ and *bla*_NDM_. To activate the CRISPR/Cas12a system, the RnP complex was formed and mixed with the RPA amplicon. The target sequence was precisely *cis*-cleaved under crRNA guidance, simultaneously generating a *trans*-cleavage of ssDNA probe (FQ-ssDNA). The fluorescence signal generated by the cleavage of the ssDNA reporter was detected using a real-time PCR instrument and through the naked eye. When both ends of the ssDNA probe were labeled with FAM and biotin, the readout was visualized using LFS for point-of-care testing (POCT). The entire procedure of the RCCS was completed within 40 min (RPA reaction, 20 min; CRISPR/Cas12a assay, 20 min).

### 3.2. Screening of RPA Primer and crRNA Sequence

**Figure 2:**
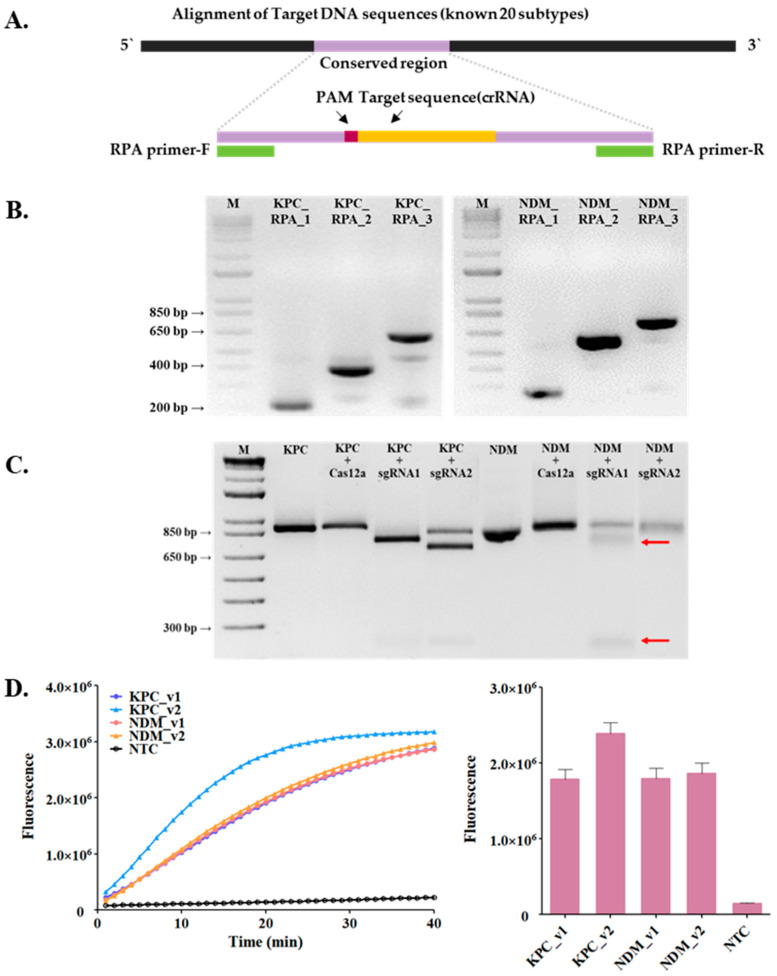
Screening of the PRA primer and crRNA sequence for *bla*_KPC_ and *bla*_NDM_ gene detection using the RCCS platform. (**A**) An overview of the crRNA and RPA primer design. (**B**) Amplification of *bla*_KPC_ (left) and *bla*_NDM_ (right) using RPA-1/2/3/ primer pairs. (**C**) *Cis*-cleavage assay using two crRNA candidates, visualizing the agarose gel electrophoresis. Red arrows point to cleaved fragments of template DNA. (**D**) *trans*-cleavage assay using two crRNA candidates and ssDNA probes. (left) Monitoring the fluorescence signal by real-time PCR and (right) endpoint fluorescence signal by UV trans-illuminator. Error bars represent standard deviation.

As shown in Figure 2B, KPC-RPA-3 (623 bp) and NDM-RPA-2 (587 bp), which were the longest among the three primer candidates, showed high efficiency of RPA reactions, making them the most suitable candidates. Subsequently, crRNAs targeting the conserved sequences of the target genes were designed based on the locations of the RPA primers (Figure 2A and Table 2). The guide RNA (gRNA) sequences complementary to the target site were designed on the conserved sequence region, including the PAM site, gRNAs, and conserved stem–loop sequence. The optimal crRNA was determined using a CRISPR/Cas12a cleavage assay using synthetic genes of *bla*_KPC_ and *bla*_NDM_. First, a *cis*-cleavage assay of LbCas12a on *bla*_KPC_ and *bla*_NDM_ was performed. As shown in Figure 2C, *bla*_KPC_ and *bla*_NDM_ were effectively cleaved by LbCas12a in both of the two crRNA candidates. We further investigated the *trans*-cleavage activity of CRISPR/Cas12a using a dually-labeled substrate ssDNA probe. The target site, including crRNA, was amplified by the RPA reaction, and the amplicons were detected by the CRISPR/Cas12a *trans*-cleavage assay. As illustrated in Figure 2D, the fluorescence signals of all crRNAs continuously increased over time. The non-template control (NTC) did not generate a fluorescence signal, and a difference was noticed in the activities of the two crRNAs. CrRNA2 demonstrated higher efficiency than crRNA1 for KPC, whereas no significant difference was noticed between the activities of crRNA1 and crRNA2 for NDM. Based on these results, we selected crRNA2 for KPC and crRNA1 for NDM or CRISPR/Cas12a-mediated detection (Figure 2D).

### 3.3. Optimization of the RCCS Conditions

The RCCS was based on RPA amplification followed by CRISPR/Cas12a-mediated detection. First, the optimal temperature and time for the RPA reaction were determined to be 10–30 min (in 10-min intervals) and a temperature range of 37–41 °C (at 2 °C intervals). Significant differences among intensities of different bands amplified at 37, 39, and 41 °C were noticed (Figure S1), and 39 °C was selected as the working temperature. In addition, the highest band intensity in RPA was observed after amplification for 20 min. Finally, the best amplification conditions for RPA were KPC-RPA-3 for *bla*_KPC_, NDM-RPA-2 for *bla*_NDM_, 39 °C, and 20 min.

**Figure 3:**
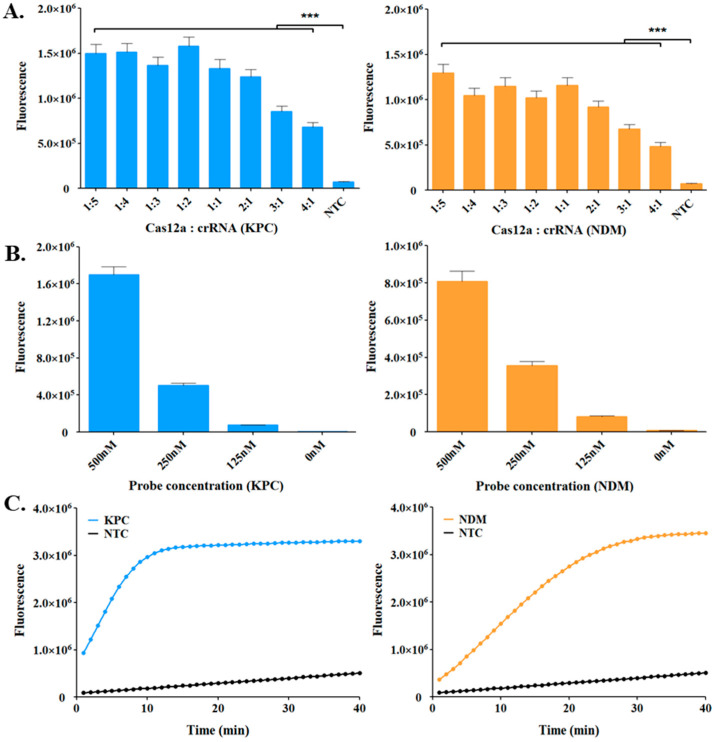
Optimization of the RCCS reaction conditions for *bla*_KPC_ and *bla*_NDM_ genes detection. (**A**) Optimization of the Cas12a:crRNA ratio. (**B**) Optimization of the concentration of ssDNA probe. The endpoint fluorescence was measured by UV trans-illuminator after 30 min of incubation at 37 °C. (**C**) Detection of *bla*_KPC_ and *bla*_NDM_ genes using RCCS platform under optimal conditions. Fluorescence signal was obtained using real-time PCR. All experiments were conducted three times, and the error bars indicate the standard deviation. Statistical analysis used the *t*-test for multiple comparisons with NTC (*** *p* < 0.001).

The CRISPR system consisted of three essential components: the Cas12a enzyme, crRNA, and ssDNA probe; we optimized each element to enhance the detection efficiency (Figure 3). First, the ratio of RnP (Cas12a:crRNA) was optimized to enhance the efficiency of the RCCS by checking the ratios of 1:5, 1:4, 1:3, 1:2, 1:1, 2:1, 3:1, and 4:1 (early RnP concentration, 175 nM). At the ratio of 1:1, the fluorescence signal decreased with increasing concentration of Cas12a, whereas no significant difference was noticed with increasing crRNA concentration. Then, we optimized the concentration of the ssDNA probe because the reporter was crucial for fluorescence detection. As shown in Figure 3B, we noticed that the fluorescence intensity increased with increasing concentrations of the probe from 125 to 500 nM. The strongest fluorescence signal was observed at 500 nM probe concentration. No significant differences were observed between the target genes (*bla*_KPC_ and *bla*_NDM_). Therefore, 500 nM was selected as the optimal ssDNA probe concentration for RCCS. Based on these results, the best conditions for detecting *bla*_KPC_ and *bla*_NDM_ genes were a Cas12a:crRNA ratio of 1:5 and ssDNA concentration of 500 nM. Subsequently, the RCCS was used to detect the *bla*_KPC_ and *bla*_NDM_ under optimal conditions, and the fluorescence signal was monitored. As shown in Figure 3C, a fluorescence signal was generated for both target genes within 15 min.

### 3.4. Specificity and Sensitivity of the RCCS

**Figure 4:**
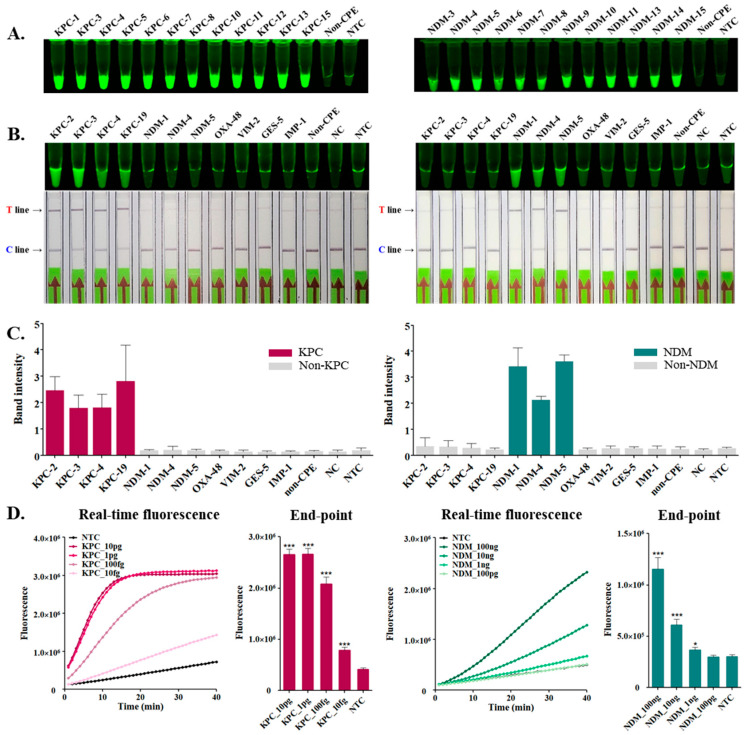
Determination of specificity and sensitivity of the RCCS platform for the detection of *bla*_KPC_ (left) and *bla*_NDM_ (right). NC, negative control; NTC, non-template control reaction. (**A**) The *trans*-cleavage activity of the RCCS platform targeting subtypes of *bla*_KPC_ and *bla*_NDM_ genes-containing plasmids. (**B**) Specificity test of the RCCS platform through endpoint imaging and LFS assay. The specificity was determined using clinical isolates, including carbapenemase genes or non-CPE strain. (**C**) The relative quantifications of band intensities obtained from the C-line and T-line of LFS. (**D**) Sensitivity test of the RCCS platform using ten-fold serially diluted plasmid containing *bla*_KPC_ and *bla*_NDM_ as a template. All experiments were conducted three times, and the error bars indicate the standard deviation. The *t*-test for multiple comparisons was used for statistical analysis (* *p* < 0.05; *** *p* < 0.001).

To evaluate the specificity of the RCCS, we used synthetic genes of *bla*_KPC_ and *bla*_NDM_ subtypes and those encoding other carbapenemases such as *bla*_OXA-48_, *bla*_VIM-2_, *bla*_GES-5_, and *bla*_IMP-1_. These synthetic genes were trans-cleaved by the RCCS and observed using a UV transilluminator for fluorescence signals from the endpoint products. Subtypes *bla*_KPC_ and *bla*_NDM_ exhibited strong fluorescence signals, and no cross-reaction for the non-CPE strain was noticed (Figure 4A). Simultaneously, the subtype genes of *bla*_KPC_ and *bla*_NDM_ were tested for comparison with other carbapenemase-encoding genes and that of a non-CPE strain. As shown in Figure 4B, strong fluorescence signals were detected for the four KPC (KPC-2, -3, -4, and-19) and three NDM (NDM-1, -4, and-5) types, whereas no fluorescence was detected for the non-CPE strain. Furthermore, we confirmed the specificity of the RCCS using LFS, which showed a distinct test line (Figure 4B). The results of three validation experiments confirmed the high specificity of the RCCS for carbapenemase-encoding genes.

The sensitivity of the RCCS was evaluated using serially diluted plasmids containing *bla*_KPC_ and *bla*_NDM_. We observed a gradual decline in fluorescence signals with decreasing concentrations of plasmids. As shown in Figure 4D, *bla*_KPC_ could be detected up to 10 fg/μL, while *bla*_NDM_ could be detected up to 1 ng/μL, indicating that the RCCS platform had a much higher sensitivity for detecting *bla*_KPC_ than for detecting *bla*_NDM_.

### 3.5. Examination of the Clinical Strains

We validated the clinical application of the RCCS for detecting nucleic acids by comparing it with conventional PCR. Eight KPC-producing and seven NDM-producing isolates showed perfect agreement between both methods. Additionally, isolates not encoding *bla*_KPC_ and *bla*_NDM_ and non-CPE isolates showed negative results, thereby demonstrating 100% concordance and confirming the high detection accuracy of the RCCS platform (Table 3).

### 3.6. Evaluation of RCCS in Spiked Sample

**Figure 5:**
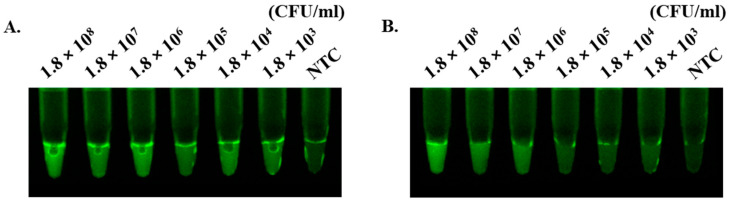
Detection of carbapenemase genes (*bla*_KPC_ and *bla*_NDM_) by using RCCS in bacteria-spiked urine samples. The endpoint fluorescence was detected by UV trans-illuminator after 30 min. (**A**) *bla*_KPC_. (**B**) *bla*_NDM_. *bla*_KPC_ was detected at levels up to 10^3^ CFU/mL, and *bla*_NDM_ was detected at levels up to 10^6^ CFU/mL.

We evaluated the detection capacity of RCCS for *bla*_KPC_ and *bla*_NDM_ genes in synthetic urine samples. As shown in Figure 5, we observed a gradual decline in fluorescence signal as the concentration decreased. The *bla*_KPC_ was detectable up to 10^3^ CFU/mL, while *bla*_NDM_ was not detected starting from 10^5^ CFU/mL (Figure 5).

## 4. Discussion

According to the KDCA report, the proportion of CPE among CRE infections in Korea has increased rapidly from 49.9% in 2018 to 71.0% in 2022. Among CPE, the KPC type accounts for 77.1%, and the NDM type accounts for 16.8%, together making up over 90% [5]. Moreover, CRE infections caused by CPE are more prevalent in long-term care hospitals and small- to medium-sized hospitals than in general hospitals, where the mortality rate can reach up to 75%, highlighting the critical need for simple and rapid detection and effective infection control [27]. This emphasizes an urgent need for the development of highly efficient and accessible diagnostic methods targeting specific genes.

The detection methods for CPE are traditional culture methods, biochemical identification, and PCR-based nucleic acid detection. However, these methods are laborious and time-consuming and require specialized equipment and skilled technicians, which are not suitable for point-of-care testing in resource-poor areas [28]. Given the limitations of the current detection techniques, developing a rapid, accurate, and field-deployable diagnostic platform is urgently required. According to the WHO (ASSURED CRITERIA), an ideal diagnostic assay for any pathogen should be inexpensive, accurate, provide rapid results, be applicable as POCT, and require little or no specialized equipment or technical support [11]. CRISPR-based technologies can potentially address these challenges. The CRISPR–Cas system offers several advantages: (i) it is simple and portable, allowing on-site detection using lateral flow analysis without the need for special equipment or a specific environment [29]; (ii) it can be combined with fluorophores, quenchers, and other agents to enable visual confirmation of the results in a short time [10]; and (iii) it shows sensitivity and specificity equal to or better than existing PCR methods, thereby significantly reducing costs [11]. Therefore, the CRISPR/Cas system has been recognized as a novel approach for next-generation molecular diagnostics [10]. In 2020, a rapid test method was developed for detecting SARS-CoV-2 infection within 40 min by combining DETECTR with LAMP and CRISPR–Cas12 [21]. In addition, a Cas13-based SHERLOCK system has been developed for detecting Zika and Dengue viruses with attomole-level sensitivity [20]. Recently, detection methods for life-threatening bacteria, such as *Neisseria gonorrhoeae*, MRSA, carbapenem-resistant *Acinetobacter baumannii*, and *Pseudomonas aeruginosa*, have been reported by integrating isothermal amplification methods with the CRISPR/Cas system [29,30,31,32].

Recently, the combination of isothermal nucleic acid amplification and CRISPR-based diagnostic technology has gained attention as an innovative tool in molecular diagnostics [33]. To enhance analytical sensitivity, isothermal amplification methods, such as LAMP and RPA, were typically used before the introduction of CRISPR [34]. LAMP has been integrated with the CRISPR/Cas12a system targeting SARS-CoV-2, and a diagnostic platform using RPA and CRISPR/Cas12b has been able to detect carbapenem-resistant *A. baumannii* by targeting *OXA-51* and *OXA-23* [32,35]. LAMP is a complex method that requires a reaction temperature of approximately 65 °C, which is much higher than that required for CRISPR-mediated reactions (37 °C). In contrast, RPA requires simple primer designing, and its optimal reaction temperature is 37–42 °C; therefore, grafting RPA onto the CRISPR system may be advantageous [29]. 

In this study, we developed a method for detecting *bla*_KPC_ and *bla*_NDM_ by combining RPA with the CRISPR/Cas12a system. Given that differences in crRNA sequences can affect the efficiency of the CRISPR/Cas12a system, selecting the proper crRNA is crucial for accurately detecting target genes [36]. Therefore, we initially designed and evaluated crRNA candidates and RPA primers and selected the optimal crRNA and RPA primer for detecting *bla*_KPC_ and *bla*_NDM_. Subsequently, we established the RCCS by combining RPA with CRISPR/Cas12a for detecting carbapenem-resistant genes and optimized its capacity using three methods: real-time fluorescence monitoring, visible endpoint fluorescence, and LFS. The platform demonstrated high sensitivity, with detection limits at attomolar levels for *bla*_KPC_ and picomolar levels for *bla*_NDM_, and achieved 100% specificity. Notably, the RCCS detected target genes within 30~40 min, significantly faster than conventional PCR methods, which typically require 90 min. Existing studies utilizing CRISPR–Cas12a systems, such as RAA- and RPA-based methods, have reported detection limits as low as 10 copies/µL with detection times ranging from 1 to 1.5 h. In contrast, our RCCS system not only demonstrated a significantly faster detection time of 30~40 min but also maintained a comparable sensitivity range of 10^3^ to 10⁶ CFU/mL (Table 4). Our findings highlight the potential of the RCCS system as a more efficient and rapid alternative for detecting AMR genes, offering distinct advantages over conventional fluorescence-based approaches. Furthermore, these results suggest that the RCCS platform could be a useful tool for the rapid and accurate detection of carbapenem-resistant genes, particularly in resource-limited settings where rapid and reliable diagnostics are essential.

We successfully established an effective diagnostic technique based on CRISPR/Cas12a combined with RPA, which can meet the need for rapid and affordable detection of carbapenem-resistant genes. However, this study has some limitations. First, the RCCS platform struggles to perform multiple reactions simultaneously, requiring individual amplification and cleavage of each carbapenem-resistant gene. Second, since the RPA and CRISPR/Cas12a systems react sequentially, background signals may arise owing to carryover contamination by opening the reaction tube. Recent studies have reported integrating DNA amplification technology with a CRISPR detection system for one-step reactions, thereby overcoming the existing limitation [40]. Thus, we aim to develop the RCCS platform-based one-step detection method and advance the development of a device capable of multiplexing for point-of-care applications in further study.

In summary, we developed a rapid, sensitive, and cost-effective RCCS platform for detecting carbapenemase genes. This platform is highly accurate and cost-effective, making it an ideal POCT solution for early diagnosis and infection control. Moreover, it has the potential for broad applications in detecting other antibiotic-resistant genes, highlighting its versatility. Although further optimization is required to address several issues, such as multiplexing capabilities and background signal reduction, the RCCS platform for detecting antimicrobial-resistant genes represents a significant advancement in molecular diagnostics, particularly in resource-limited environments.

## Figures and Tables

**Table 1:** crRNAs used in this study.

Name	Sequence (5′–3′)	PAM
gLb_KPC_v1	CGAGGUUGGUCAGCGCGGUGGCA **AUCUACACUUAGUAGAAAUUCCC**	TTTC
gLb_KPC_v2	CGCGUACACACCGAUGGAGCCGC **AUCUACACUUAGUAGAAAUUCCC**	TTTG
gLb_NDM_v1	GCUGUCCUUGAUCAGGCAGCCAC **AUCUACACUUAGUAGAAAUUCCC**	TTTG
gLb_NDM_v2	GCUGGCCUUGGGGAACGCCGCAC **AUCUACACUUAGUAGAAAUUCCC**	TTTG

Underlined: Target sequence, Bold: crRNA scaffold sequence for Cas12a protein.

**Table 2:** RPA primers used in this study.

Name (Product Size)	Region	Sequence (5′–3′)
KPC_RPA_1	F(17-)	GTCTAGTTCTGCTGTCTTGTCTCTCATGGC
(201bp)	R(-217)	CCTTGAATGAGCTGCACAGTGGGAAGCGCT
KPC_RPA_2	F(17-)	GTCTAGTTCTGCTGTCTTGTCTCTCATGGC
(384bp)	R(-400)	CGGCGGCGTTATCACTGTATTGCACGGCGG
KPC_RPA_3	F(17-)	GTCTAGTTCTGCTGTCTTGTCTCTCATGGC
(623bp)	R(-639)	GTTTCCCTTTAGCCAATCAACAAACTGCTG
NDM_RPA_1	F(525-)	CAACTTTGGCCCGCTCAAGGTATTTTACCC
(262bp)	R(-786)	CGTATGAGTGATTGCGGCGCGGCTATCGGG
NDM_RPA_2	F(168-)	GAATGTCTGGCAGCACACTTCCTATCTCGA
(587bp)	R(-754)	CGGAATGGCTCATCACGATCATGCTGGCCT
NDM_RPA_3	F(9-)	GCCCAATATTATGCACCCGGTCGCGAAGCT
(746bp)	R(-754)	CGGAATGGCTCATCACGATCATGCTGGCCT

**Table 3:** Prevalence of *bla*_KPC_ and *bla*_NDM_ genes in clinical isolates of *Enterobacterales* using RCCS assay and PCR.

RCCS Assay	PCR			Total
	*bla*_KPC_ (n)	*bla*_NDM_ (n)	Negative	
*bla*_KPC_ (n)	8	0	0	8
*bla*_NDM_ (n)	0	7	0	7
Negative	0	0	10	10
Total	8	7	10	25

**Table 4:** Application of the CRISPR-based tools for pathogen detection.

Cas Protein	Pathogen	Amplification Methods	Detection	Platform	Sensitivity	Time	Ref.
Cas12a	Methicillin-resistant *S. aureus* (MRSA)	RAA	Fluorescence	RAA-Cas12a	10 copies/µL	1 h	[37]
Cas12a	Pathogenic *Yersinia enterocolitica*	RPA	Fluorescence	CRISPR/Cas12a-RPA	1.7 CFU/mL	45 min	[38]
Cas12a	Carbapenem-resistant *A. baumanii* (CRAB)	RPA	Fluorescence	RPA– CRISPR–Cas12a	1.3 × 10^−6^ ng/µL	90 min	[32]
Cas13a	Carbapenem-resistant pathogens	LAMP	LFS	LAMP– CRISPR–13a	10^3^ CFU/mL ~10^7^ CFU/mL	2 h	[39]
Cas12a	Carbapenem-resistant pathogens	RPA	Fluorescence, LFS	RCCS	10^3^ CFU/mL ~10^6^ CFU/mL	30~40 min	This study

## Data Availability

All data generated or analyzed during this study are included in this published article and its Supplementary Information Files.

## Supplementary Materials

The following supporting information can be downloaded at: https://www.mdpi.com/article/10.3390/bios14110536/s1. Figure S1: Agarose gel electrophoresis results for optimizing RPA conditions. Agarose gel electrophoresis results of amplification usingRPA-1/2/3 primer pairs. (A. temperature and B. time)**.** Figure S2: Location of crRNAs within the conserved region of *bla*_KPC_. A. gLb_KPC_v1. B. gLb_KPC_v2. Numbers at the top of the figure denote the positions within the sequence. Figure S3: Location of crRNAs within the conserved region of bla_NDM_. A. gLb_NDM_v1. B. gLb_NDM_v2. Numbers at the top of the figure denote the positions within the sequence.
