# Supplementary material for: Development of a Recombinase Polymerase Amplification-Coupled CRISPR/Cas12a Platform for Rapid Detection of Antimicrobial-Resistant Genes in Carbapenem-Resistant Enterobacterales
Source: Biosensors (Basel). 2024 Nov 5;14(11):536. doi: 10.3390/bios14110536 (PMC11591667; doi:10.3390/bios14110536)
Supplement: Supplementary file 1 [file biosensors-14-00536-s001.zip › biosensors-3235690 - supplementary.pdf]

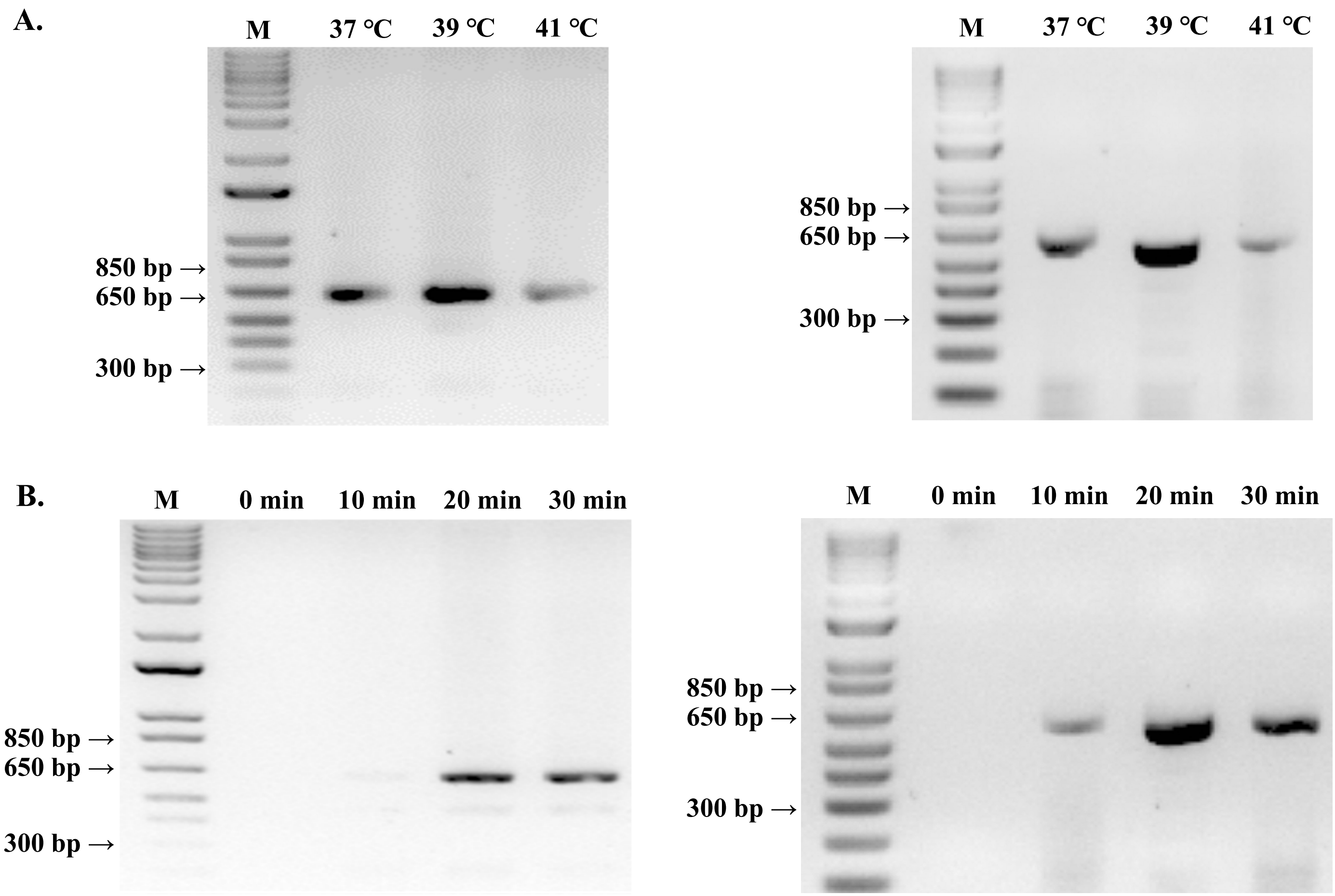


**Supplementary Figure S1.** Agarose gel electrophoresis results for optimizing RPA conditions. Agarose gel electrophoresis results of amplification usingRPA-1/2/3 primer pairs. (A. temperature and B. time).

**A.**

| | | | 40 | 41 | 42 | 43 | 44 | 45 | 46 | 47 | 48 | 49 | 50 | 51 | 52 | 53 | 54 | 55 | 56 | 57 | 58 | 59 | 60 | 61 | 62 | 63 | 64 | 65 | 66 | 67 | 68 | 69 | 70 | 71 | 72 | 73 | 74 | 75 | 76 | 77 | 78 | 79 | 80 | 81 | 82 | 83 | 84 | 85 | 86 | 87 | 88 | 89 | 90 | 91 | 92 | 93 | 94 | 95 |
|---|---|---|---|---|---|---|---|---|---|---|---|---|---|---|---|---|---|---|---|---|---|---|---|---|---|---|---|---|---|---|---|---|---|---|---|---|---|---|---|---|---|---|---|---|---|---|---|---|---|---|---|---|---|---|---|---|---|---|
| Consensus | | | T | C | A | T | G | G | C | C | G | C | T | G | G | C | T | G | G | C | T | T | T | T | C | T | G | C | C | A | C | C | G | C | G | C | T | G | A | C | C | A | A | C | C | T | C | G | T | C | G | C | G | G | A | A | C | C |
| KPC-2 | AY034847:5-887 | Carbapenem;Cephalosp | T | C | A | T | G | G | C | C | G | C | T | G | G | C | T | G | G | C | T | T | T | T | C | T | G | C | C | A | C | C | G | C | G | C | T | G | A | C | C | A | A | C | C | T | C | G | T | C | G | C | G | G | A | A | C | C |
| KPC-3 | AF395881:0-882 | Carbapenem;Cephalosp | T | C | A | T | G | G | C | C | G | C | T | G | G | C | T | G | G | C | T | T | T | T | C | T | G | C | C | A | C | C | G | C | G | C | T | G | A | C | C | A | A | C | C | T | C | G | T | C | G | C | G | G | A | A | C | C |
| KPC-4 | EU447304.1:14-896 | Carbapenem;Cephalosp | T | C | A | T | G | G | C | C | G | C | T | G | G | C | T | G | G | C | T | T | T | T | C | T | G | C | C | A | C | C | G | C | G | C | T | G | A | C | C | A | A | C | C | T | C | G | T | C | G | C | G | G | A | A | C | C |
| KPC-5 | EU400222:2159-3041 | Carbapenem;Cephalosp | T | C | A | T | G | G | C | C | G | C | T | G | G | C | T | G | G | C | T | T | T | T | C | T | G | C | C | A | C | C | G | C | G | C | T | G | A | C | C | A | A | C | C | T | C | G | T | C | G | C | G | G | A | A | C | C |
| KPC-6 | EU555534:0-882 | Carbapenem;Cephalosp | T | C | A | T | G | G | C | C | G | C | T | G | G | C | T | G | G | C | T | T | T | T | C | T | G | C | C | A | C | C | G | C | G | C | T | G | A | C | C | A | A | C | C | T | C | G | T | C | G | C | G | G | A | A | C | C |
| KPC-7 | EU729727:0-882 | Carbapenem;Cephalosp | T | C | A | T | G | G | C | C | G | C | T | G | G | C | T | G | G | C | T | T | T | T | C | T | G | C | C | A | C | C | G | C | G | C | T | G | A | C | C | A | A | C | C | T | C | G | T | C | G | C | G | G | A | A | C | C |
| KPC-8 | FJ234412:0-882 | Carbapenem;Cephalosp | T | C | A | T | G | G | C | C | G | C | T | G | G | C | T | G | G | C | T | T | T | T | C | T | G | C | C | A | C | C | G | C | G | C | T | G | A | C | C | A | A | C | C | T | C | G | T | C | G | C | G | G | A | A | C | C |
| KPC-10 | GQ140348:0-882 | Carbapenem;Cephalosp | T | C | A | T | G | G | C | C | G | C | T | G | G | C | T | G | G | C | T | T | T | T | C | T | G | C | C | A | C | C | G | C | G | C | T | G | A | C | C | A | A | C | C | T | C | G | T | C | G | C | G | G | A | A | C | C |
| KPC-11 | HM066995:0-882 | Carbapenem;Cephalosp | T | C | A | T | G | G | C | C | G | C | T | G | G | C | T | G | G | C | T | T | T | T | C | T | G | C | C | A | C | C | G | C | G | C | T | G | A | C | C | A | A | C | C | T | C | G | T | C | G | C | G | G | A | A | C | C |
| KPC-12 | HQ641421:0-882 | Carbapenem;Cephalosp | T | C | A | T | G | G | C | C | G | C | T | G | G | C | T | G | G | C | T | T | T | T | C | T | G | C | C | A | C | C | G | C | G | C | T | G | A | C | C | A | A | C | C | T | C | G | T | C | G | C | G | G | A | A | C | C |
| KPC-13 | HQ342889:0-882 | Carbapenem;Cephalosp | T | C | A | T | G | G | C | C | G | C | T | G | G | C | T | G | G | C | T | T | T | T | C | T | G | C | C | A | C | C | G | C | G | C | T | G | A | C | C | A | A | C | C | T | C | G | T | C | G | C | G | G | A | A | C | C |
| KPC-15 | KC433553:0-882 | Carbapenem;Cephalosp | T | C | A | T | G | G | C | C | G | C | T | G | G | C | T | G | G | C | T | T | T | T | C | T | G | C | C | A | C | C | G | C | G | C | T | G | A | C | C | A | A | C | C | T | C | G | T | C | G | C | G | G | A | A | C | C |
| KPC-16 | KC465199:0-882 | Carbapenem;Cephalosp | T | C | A | T | G | G | C | C | G | C | T | G | G | C | T | G | G | C | T | T | T | T | C | T | G | C | C | A | C | C | G | C | G | C | T | G | A | C | C | A | A | C | C | T | C | G | T | C | G | C | G | G | A | A | C | C |
| KPC-17 | KC465200:0-882 | Carbapenem;Cephalosp | T | C | A | T | G | G | C | C | G | C | T | G | G | C | T | G | G | C | T | T | T | T | C | T | G | C | C | A | C | C | G | C | G | C | T | G | A | C | C | A | A | C | C | T | C | G | T | C | G | C | G | G | A | A | C | C |
| KPC-19 | KJ775801:0-882 | Carbapenem;Cephalosp | T | C | A | T | G | G | C | C | G | C | T | G | G | C | T | G | G | C | T | T | T | T | C | T | G | C | C | A | C | C | G | C | G | C | T | G | A | C | C | A | A | C | C | T | C | G | T | C | G | C | G | G | A | A | C | C |
| KPC-22 | KM379100:0-882 | Carbapenem;Cephalosp | T | C | A | T | G | G | C | C | G | C | T | G | G | C | T | G | G | C | T | T | T | T | C | T | G | C | C | A | C | C | G | C | G | C | T | G | A | C | C | A | A | C | C | T | C | G | T | C | G | C | G | G | A | A | C | C |
| KPC-24 | KR052099.1:5211-6093 | Carbapenem;Cephalo | T | C | A | T | G | G | C | C | G | C | T | G | G | C | T | G | G | C | T | T | T | T | C | T | G | C | C | A | C | C | G | C | G | C | T | G | A | C | C | A | A | C | C | T | C | G | T | C | G | C | G | G | A | A | C | C |
| sgRNA | | | | | | | | | | | | | | | | | | | | | | PAM | | | | gAs_KPC_v1/gLB_KPC_v1 | | | | | | | | | | | | | | | | | | | | | | | | | | | | | | | | | | | | |

**B.**

| | | | 116 | 117 | 118 | 119 | 120 | 121 | 122 | 123 | 124 | 125 | 126 | 127 | 128 | 129 | 130 | 131 | 132 | 133 | 134 | 135 | 136 | 137 | 138 | 139 | 140 | 141 | 142 | 143 | 144 | 145 | 146 | 147 | 148 | 149 | 150 | 151 | 152 | 153 | 154 | 155 | 156 | 157 | 158 | 159 | 160 | 161 | 162 | 163 | 164 | 165 | 166 | 167 | 168 | 169 | 170 | 171 | 172 |
|---|---|---|---|---|---|---|---|---|---|---|---|---|---|---|---|---|---|---|---|---|---|---|---|---|---|---|---|---|---|---|---|---|---|---|---|---|---|---|---|---|---|---|---|---|---|---|---|---|---|---|---|---|---|---|---|---|---|---|---|
| Consensus | | | A | C | T | T | T | G | G | C | G | G | C | T | C | C | A | T | C | G | G | T | G | T | G | T | A | C | G | C | G | A | T | G | G | A | T | A | C | C | G | G | C | T | C | A | G | G | C | G | C | A | A | C | T | G | T | A | A |
| KPC-2 | AY034847:5-887 | Carbapenem;Cephalosp | A | C | T | T | T | G | G | C | G | G | C | T | C | C | A | T | C | G | G | T | G | T | G | T | A | C | G | C | G | A | T | G | G | A | T | A | C | C | G | G | C | T | C | A | G | G | C | G | C | A | A | C | T | G | T | A | A |
| KPC-3 | AF395881:0-882 | Carbapenem;Cephalosp | A | C | T | T | T | G | G | C | G | G | C | T | C | C | A | T | C | G | G | T | G | T | G | T | A | C | G | C | G | A | T | G | G | A | T | A | C | C | G | G | C | T | C | A | G | G | C | G | C | A | A | C | T | G | T | A | A |
| KPC-4 | EU447304.1:14-896 | Carbapenem;Cephalosp | A | C | T | T | T | G | G | C | G | G | C | T | C | C | A | T | C | G | G | T | G | T | G | T | A | C | G | C | G | A | T | G | G | A | T | A | C | C | G | G | C | T | C | A | G | G | C | G | C | A | A | C | T | G | T | A | A |
| KPC-5 | EU400222:2159-3041 | Carbapenem;Cephalosp | A | C | T | T | T | G | G | C | G | G | C | T | C | C | A | T | C | G | G | T | G | T | G | T | A | C | G | C | G | A | T | G | G | A | T | A | C | C | G | G | C | T | C | A | G | G | C | G | C | A | A | C | T | G | T | A | A |
| KPC-6 | EU555534:0-882 | Carbapenem;Cephalosp | A | C | T | T | T | G | G | C | G | G | C | T | C | C | A | T | C | G | G | T | G | T | G | T | A | C | G | C | G | A | T | G | G | A | T | A | C | C | G | G | C | T | C | A | G | G | C | G | C | A | A | C | T | G | T | A | A |
| KPC-7 | EU729727:0-882 | Carbapenem;Cephalosp | A | C | T | T | T | G | G | C | G | G | C | T | C | C | A | T | C | G | G | T | G | T | G | T | A | C | G | C | G | A | T | A | G | A | T | A | C | C | G | G | C | T | C | A | G | G | C | G | C | A | A | C | T | G | T | A | A |
| KPC-8 | FJ234412:0-882 | Carbapenem;Cephalosp | A | C | T | T | T | G | G | C | G | G | C | T | C | C | A | T | C | G | G | T | G | T | G | T | A | C | G | C | G | A | T | G | G | A | T | A | C | C | G | G | C | T | C | A | G | G | C | G | C | A | A | C | T | G | T | A | A |
| KPC-10 | GQ140348:0-882 | Carbapenem;Cephalosp | A | C | T | T | T | G | G | C | G | G | C | T | C | C | A | T | C | G | G | T | G | T | G | T | A | C | G | C | G | A | T | G | G | A | T | A | C | C | G | G | C | T | C | A | G | G | C | G | C | A | A | C | T | G | T | A | A |
| KPC-11 | HM066995:0-882 | Carbapenem;Cephalosp | A | C | T | T | T | G | G | C | G | G | C | T | C | C | A | T | C | G | G | T | G | T | G | T | A | C | G | C | G | A | T | G | G | A | T | A | C | C | G | G | C | T | C | A | G | G | C | G | C | A | A | C | T | G | T | A | A |
| KPC-12 | HQ641421:0-882 | Carbapenem;Cephalosp | A | C | T | T | T | G | G | C | G | G | C | T | C | C | A | T | C | G | G | T | G | T | G | T | A | C | G | C | G | A | T | G | G | A | T | A | C | C | G | G | C | T | C | A | G | G | C | G | C | A | A | C | T | G | T | A | A |
| KPC-13 | HQ342889:0-882 | Carbapenem;Cephalosp | A | C | T | T | T | G | G | C | G | G | C | T | C | C | A | T | C | G | G | T | G | T | G | T | A | C | G | C | G | A | T | G | G | A | T | A | C | C | G | G | C | T | C | A | G | G | C | G | C | A | A | C | T | G | T | A | A |
| KPC-15 | KC433553:0-882 | Carbapenem;Cephalosp | A | C | T | T | T | G | G | C | G | G | C | T | C | C | A | T | C | G | G | T | G | T | G | T | A | C | G | C | G | A | T | G | G | A | T | A | C | C | G | G | C | T | C | A | G | G | C | G | C | A | A | C | T | G | T | A | A |
| KPC-16 | KC465199:0-882 | Carbapenem;Cephalosp | A | C | T | T | T | G | G | C | G | G | C | T | C | C | A | T | C | G | G | T | G | T | G | T | A | C | G | C | G | A | T | G | G | A | T | A | C | C | G | G | T | T | C | A | G | G | C | G | C | A | A | C | T | G | T | A | A |
| KPC-17 | KC465200:0-882 | Carbapenem;Cephalosp | A | C | T | T | T | G | G | C | G | G | C | T | C | C | A | T | C | G | G | T | G | T | G | T | A | C | G | C | G | A | T | G | G | A | T | A | C | C | G | G | C | T | C | A | G | G | C | G | C | A | A | C | T | G | T | A | A |
| KPC-19 | KJ775801:0-882 | Carbapenem;Cephalosp | A | C | T | T | T | G | G | C | G | G | C | T | C | C | A | T | C | G | G | T | G | T | G | T | A | C | G | C | G | A | T | G | G | A | T | A | C | C | G | G | C | T | C | A | G | G | C | G | C | A | A | C | T | G | T | A | A |
| KPC-22 | KM379100:0-882 | Carbapenem;Cephalosp | A | C | T | T | T | G | G | C | G | G | C | T | C | C | A | T | C | G | G | T | G | T | G | T | A | C | G | C | G | A | T | G | G | A | T | A | C | C | G | G | C | T | C | A | G | G | C | G | C | A | A | C | T | G | T | A | A |
| KPC-24 | KR052099.1:5211-6093 | Carbapenem;Cephalo | A | C | T | T | T | G | G | C | G | G | C | T | C | C | A | T | C | G | G | T | G | T | G | T | A | C | G | C | G | A | T | G | G | A | T | A | C | C | G | G | C | T | C | A | G | G | C | G | C | A | A | C | T | G | T | A | A |
| sgRNA | | | | | PAM | | | | gAs_KPC_v2/gLb_KPC_v2 | | | | | | | | | | | | | | | | | | | | | | | | | | | | | | | | | | | | | | | | | | | | | | | | | |

**Figure S2.** Location of crRNAs within the conserved region of $bla_{KPC}$. **A.** gLb_KPC_v1. **B.** gLb_KPC_v2. Numbers at the top of the figure denote the positions within the sequence.

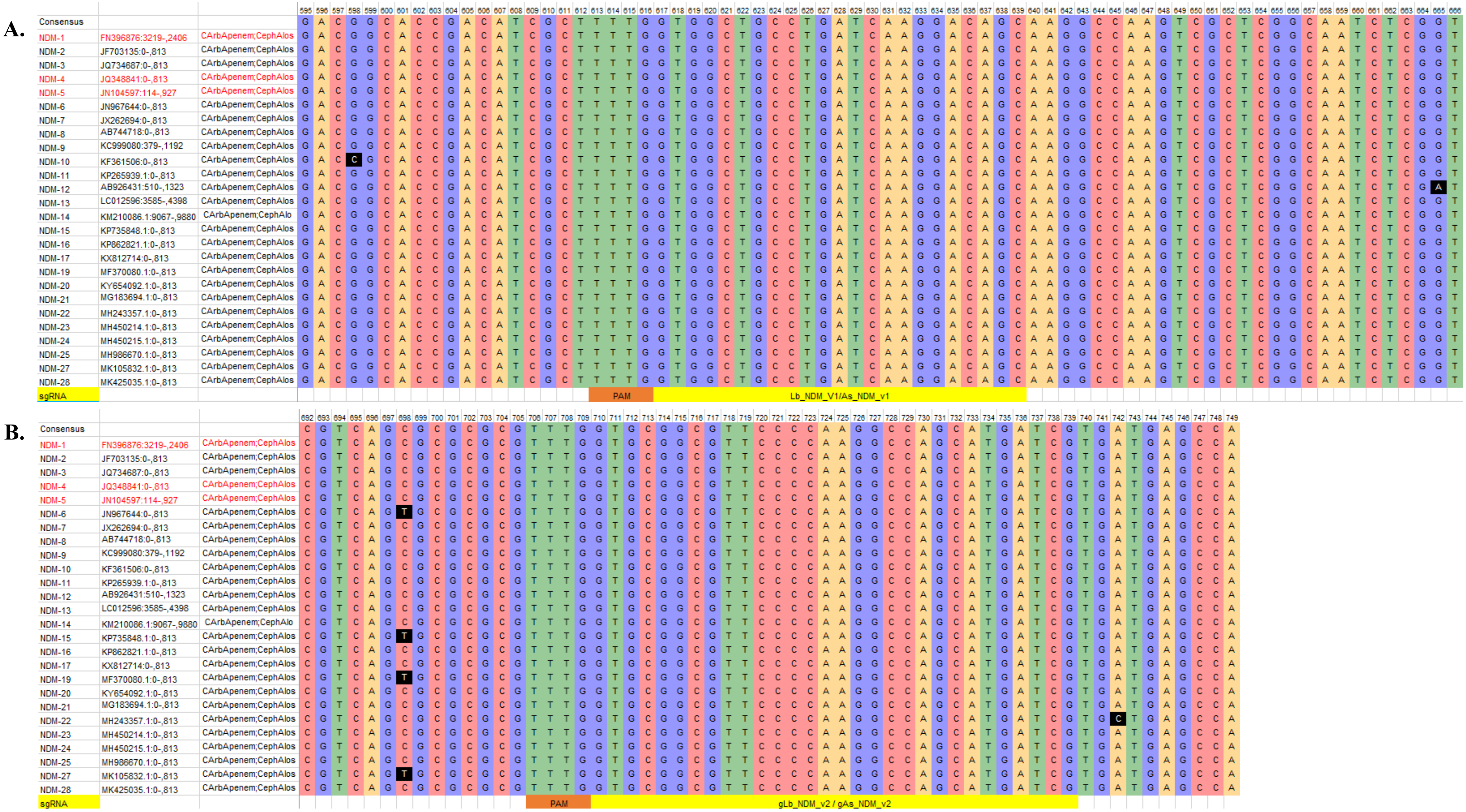


**Figure S3.** Location of crRNAs within the conserved region of $bla_{NDM}$. **A.** gLb_NDM_v1. **B.** gLb_NDM_v2. Numbers at the top of the figure denote the positions within the sequence.
